# Iowa Medical Journal

**Published:** 1853-10

**Authors:** 


### IOWA MEDICAL JOURNAL.

The first number of this periodical, devoted to medical science, was issued in August, and contains much useful and interesting matter. It is designed to be an exponent of western diseases and practice, and is to be issued monthly at "two dollars annually in advance." It is issued under the auspices of the Faculty of the Medical College at Keokuk, or the College of Physicians and Surgeons of the Iowa University, which has recently been re-organized by the appointment of an efficient Board of Professors.

The Iowa Medical Journal is under the editorial supervision of D. L. McGugin, M. D., to whom all letters on business should be addressed.

